# LiHo(PO_3_)_4_

**DOI:** 10.1107/S1600536809001767

**Published:** 2009-01-23

**Authors:** Emna Ben Zarkouna, Ahmed Driss, Mokhtar Férid

**Affiliations:** a Laboratoire de Matériaux et Cristallochimie, Faculté des Sciences, Université Tunis-El Manar, 2092 El Manar, Tunis, Tunisia; b Unité de Matériaux de Terres Rares, Centre National de Recherches en Sciences des Matériaux, BP 95 Hammam-Lif, Tunis 2050, Tunisia

## Abstract

Lithium holmium(III) polyphosphate(V), LiHo(PO_3_)_4_, belongs to the type I of polyphosphates with general formula *ALn*(PO_3_)_4_, where *A* is a monovalent cation and *Ln* is a trivalent rare earth cation. In the crystal structure, the polyphosphate chains spread along the *b*-axis direction, with a repeat period of four tetra­hedra and 2_1_ inter­nal symmetry. The Li and Ho atoms are both located on twofold rotation axes and are surrounded by four and eight O atoms, leading to a distorted tetra­hedral and dodeca­hedral coordination, respectively. The HoO_8_ polyhedra are isolated from each other, the closest Ho⋯Ho distance being 5.570 (1) Å.

## Related literature

For isotypic Li*Ln*(PO_3_)_4_ structures, see: Ben Zarkouna & Driss (2004 ▶) for *Ln* = Yb; Ben Zarkouna *et al.* (2005 ▶) for *Ln* = Er; Ben Zarkouna *et al.* (2007 ▶) for *Ln* = Tb. For related structures, see: Amami *et al.* (2004 ▶) (NaHo(PO_3_)_4_); Palkina *et al.* (1976 ▶) (*β*-KHo(PO_3_)_4_); Tranqui *et al.* (1972 ▶) (HoP_5_O_14_). For general background, see: Brown & Altermatt (1985 ▶); Durif (1995 ▶); Liu *et al.* (1999 ▶); Palkina *et al.* (1981 ▶); Inter­national Tables for X-Ray Crystallography (1968 ▶).

## Experimental

### 

#### Crystal data

LiHo(PO_3_)_4_
*M* *_r_* = 487.75
Monoclinic, 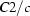

*a* = 16.280 (3) Å
*b* = 7.039 (2) Å
*c* = 9.561 (2) Å
β = 125.99 (2)°
*V* = 886.5 (4) Å^3^
*Z* = 4
Mo *K*α radiation
μ = 9.72 mm^−1^
*T* = 293 (2) K
0.31 × 0.14 × 0.07 mm

#### Data collection

Enraf–Nonius CAD-4 diffractometer
Absorption correction: ψ scan (North *et al.*, 1968 ▶) *T*_min_ = 0.095, *T*_max_ = 0.378 (expected range = 0.128–0.507)
1500 measured reflections
1078 independent reflections
1076 reflections with *I* > 2σ(*I*)
*R*_int_ = 0.016
2 standard reflections frequency: 120 min intensity decay: none

#### Refinement

*R*[*F* ^2^ > 2σ(*F* ^2^)] = 0.025
*wR*(*F* ^2^) = 0.071
*S* = 1.12
1078 reflections
84 parameters
Δρ_max_ = 2.75 e Å^−3^
Δρ_min_ = −3.18 e Å^−3^

### 

Data collection: *CAD-4 EXPRESS* (Duisenberg, 1992 ▶; Macíček & Yordanov, 1992 ▶); cell refinement: *CAD-4 EXPRESS*; Macíček & Yordanov, 1992 ▶); data reduction: *XCAD4* (Harms & Wocadlo, 1995 ▶); program(s) used to solve structure: *SHELXS97* (Sheldrick, 2008 ▶); program(s) used to refine structure: *SHELXL97* (Sheldrick, 2008 ▶); molecular graphics: *DIAMOND* (Brandenburg, 1998 ▶); software used to prepare material for publication: *SHELXL97*.

## Supplementary Material

Crystal structure: contains datablocks I, global. DOI: 10.1107/S1600536809001767/wm2216sup1.cif

Structure factors: contains datablocks I. DOI: 10.1107/S1600536809001767/wm2216Isup2.hkl

Additional supplementary materials:  crystallographic information; 3D view; checkCIF report

## Figures and Tables

**Table 1:** Selected bond lengths (Å)

Li—O2^i^	1.962 (9)
Li—O6	1.999 (9)
Ho—O3	2.287 (3)
Ho—O1^i^	2.348 (4)
Ho—O2^ii^	2.411 (3)
Ho—O6	2.516 (3)
P1—O1	1.488 (4)
P1—O6	1.499 (3)
P1—O4	1.589 (4)
P1—O5^iii^	1.594 (3)
P2—O2	1.486 (3)
P2—O3	1.488 (3)
P2—O5	1.588 (3)
P2—O4	1.601 (4)

Symmetry codes: (i) 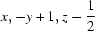; (ii) 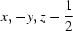; (iii) 
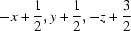
.

**Fig. 1.**
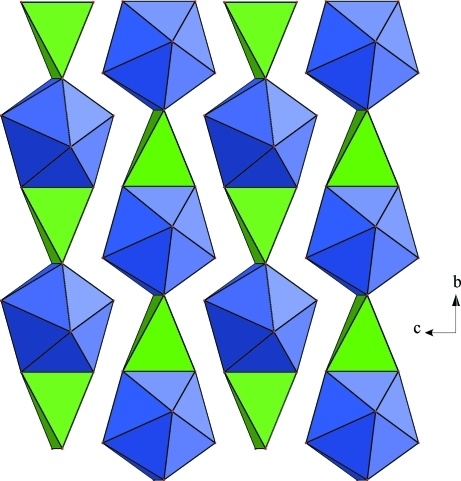
: Alternating arrangement of HoO8 and LiO4 polyhedra projected along the a axis. PO4 tetrahedra are omitted for clarity.

**Fig. 2.**
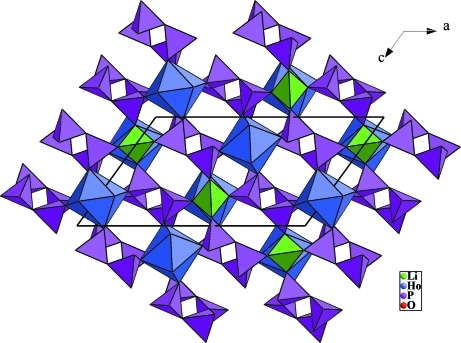
: Projection of the LiHo(PO3)4 structure along the b axis.

## supplementary crystallographic information

### Comment

The present paper is an extension of our earlier work on polyphosphates of general formula LiLn(PO_3_)_4_ [Ln = Yb (Ben Zarkouna & Driss, 2004), Er (Ben Zarkouna *et al.*, 2005) and Tb (Ben Zarkouna *et al.*, 2007)]. This family is now expanded to include the lithium holmium polyphosphate, LiHo(PO_3_)_4_, which might be of particular interest in the area of luminescent materials, because an avalanche up-conversion emission was observed previously for Ho^3+^-containing compounds (Liu *et al.*, 1999).

The LiLn(PO_3_)_4_ polyphosphates are isostructural and belong to form I according to the classification of Palkina *et al.* (1981). In this type of arrangement, helical ribbons, _∞_(PO_3_)_n_, formed by corner-sharing of PO_4_ tetrahedra, spread parallel to the *b* axis. The period of the chains corresponds to four tetrahedra but, owing to the presence of 2_1_ screw axes, only two of them are crystallographically independent. The P—O bonds involving terminal O atoms are the shortest within a PO_4_ tetrahedron, because of the dπ-pπ orbital overlap (Durif, 1995). In LiHo(PO_3_)_4_, the mean P—O bond lengths for terminal and bridging O atoms, are of 1.490 and 1.593 Å, respectively. The O—P—O angles are within the range 101.2 (2)–119.6 (2) °, the smallest and the largest ones are those involving the longest and the shortest P—O bonds, respectively. The P—O—P bridges exhibit angles of 131.6 (2) and 134.8 (2)°, larger than the O—P—O angles. The Li and Ho atoms are arranged alternately on twofold rotation axes at roughly similar spacings [3.486 (1) and 3.553 (1) Å] in comparison with the quite different spacings between K and Ho atoms [3.721 (2) and 8.589 (2) Å] in *β*-KHo(PO_3_)_4_ (Palkina *et al.*, 1976). The Ho atom is surrounded by eight terminal oxygen atoms forming a distorted dodecahedron, with an average Ho—O distance of 2.390 Å, while the Li atom is located inside an irregular tetrahedron with a mean Li—O distance of 1.980 Å. All these bond lengths are conform with those mentioned in the literature (International Tables for X-Ray Crystallography, 1968; Durif, 1995; Amami *et al.*, 2004).

By sharing edges, the HoO_8_ and LiO_4_ polyhedra are joined to produce infinite linear chains running along the *b* axis (Fig. 1), in contrast to crystal structure of NaHo(PO_3_)_4_ (form II) (Amami *et al.*, 2004) where zigzag chains of face-sharing HoO_8_ and NaO_8_ polyhedra are observed. As shown in Fig. 2, each chain shares corners of its polyhedra with four adjacent polyphosphate chains.

It is noteworthy that no O atom is common to two Ho atoms, and the closest Ho···Ho distance of 5.570 (1) Å in LiHo(PO_3_)_4_ is comparable with that found in HoP_5_O_14_ (Tranqui *et al.*, 1972). Bond-valence-sum values (Brown & Altermatt, 1985) are 0.998, 3.054, 4.936 and 4.967 valence units for Li, Ho, P1 and P2, respectively, in good agreement with the expected formal charges.

### Experimental

The title compound was prepared in single crystalline form using the flux method. At room temperature, 3 g of Li_2_CO_3_ and 0.5 g of Ho_2_O_3_ were slowly added to 10 ml of H_3_PO_4_ (85%_wt_) in a glassy carbon crucible. The resulting mixture was then progressively heated to 573 K and kept at this temperature for 8 days. After cooling to room temperature and removal of the excess phosphoric flux with boiling water, pale yellow crystals of LiHo(PO_3_)_4_ were separated.

### Refinement

The highest peak is located 0.87 Å from Ho and the deepest hole is located 1.00 Å from the same atom.

### Crystal data

**Table:**

LiHo(PO_3_)_4_	*F*(000) = 904
*M**_r_* = 487.75	*D*_x_ = 3.654 Mg m^−^^3^
Monoclinic, *C*2/*c*	Mo *K*α radiation, λ = 0.71069 Å
Hall symbol: -C 2yc	Cell parameters from 25 reflections
*a* = 16.280 (3) Å	θ = 10.1–14.7°
*b* = 7.039 (2) Å	µ = 9.72 mm^−^^1^
*c* = 9.561 (2) Å	*T* = 293 K
β = 125.99 (2)°	Plate, pale yellow
*V* = 886.5 (4) Å^3^	0.31 × 0.14 × 0.07 mm
*Z* = 4	

### Data collection

**Table:**

Enraf–Nonius CAD-4 diffractometer	1076 reflections with *I* > 2σ(*I*)
Radiation source: fine-focus sealed tube	*R*_int_ = 0.016
graphite	θ_max_ = 28.0°, θ_min_ = 3.1°
ω/2θ scans	*h* = −21→17
Absorption correction: ψ scan (North *et al.*, 1968)	*k* = −2→9
*T*_min_ = 0.095, *T*_max_ = 0.378	*l* = 0→12
1500 measured reflections	2 standard reflections every 120 min
1078 independent reflections	intensity decay: none

### Refinement

**Table:**

Refinement on *F*^2^	Primary atom site location: heavy method
Least-squares matrix: full	Secondary atom site location: difference Fourier map
*R*[*F*^2^ > 2σ(*F*^2^)] = 0.025	*w* = 1/[σ^2^(*F*_o_^2^) + (0.0459*P*)^2^ + 12.7265*P*] where *P* = (*F*_o_^2^ + 2*F*_c_^2^)/3
*wR*(*F*^2^) = 0.071	(Δ/σ)_max_ < 0.001
*S* = 1.12	Δρ_max_ = 2.75 e Å^−^^3^
1078 reflections	Δρ_min_ = −3.18 e Å^−^^3^
84 parameters	Extinction correction: *SHELXL97* (Sheldrick, 2008), Fc^*^=kFc[1+0.001xFc^2^λ^3^/sin(2θ)]^-1/4^
0 restraints	Extinction coefficient: 0.0014 (3)

### Special details

**Table:**

Geometry. All e.s.d.'s (except the e.s.d. in the dihedral angle between two l.s. planes) are estimated using the full covariance matrix. The cell e.s.d.'s are taken into account individually in the estimation of e.s.d.'s in distances, angles and torsion angles; correlations between e.s.d.'s in cell parameters are only used when they are defined by crystal symmetry. An approximate (isotropic) treatment of cell e.s.d.'s is used for estimating e.s.d.'s involving l.s. planes.
Refinement. Refinement of *F*^2^ against ALL reflections. The weighted *R*-factor *wR* and goodness of fit *S* are based on *F*^2^, conventional *R*-factors *R* are based on *F*, with *F* set to zero for negative *F*^2^. The threshold expression of *F*^2^ > σ(*F*^2^) is used only for calculating *R*-factors(gt) *etc*. and is not relevant to the choice of reflections for refinement. *R*-factors based on *F*^2^ are statistically about twice as large as those based on *F*, and *R*- factors based on ALL data will be even larger.

### Fractional atomic coordinates and isotropic or equivalent isotropic displacement parameters (Å^2^)

**Table:**

	*x*	*y*	*z*	*U*_iso_*/*U*_eq_	
Li	0.0000	0.708 (2)	0.2500	0.015 (3)	
Ho	0.0000	0.20312 (4)	0.2500	0.0073 (1)	
P1	0.13774 (8)	0.5517 (2)	0.6157 (1)	0.0067 (2)	
P2	0.14654 (8)	0.1504 (2)	0.6961 (1)	0.0068 (2)	
O1	0.1137 (3)	0.7154 (5)	0.6843 (5)	0.0118 (7)	
O2	0.0711 (3)	0.0845 (5)	0.7255 (5)	0.0116 (6)	
O3	0.1280 (3)	0.1150 (5)	0.5262 (4)	0.0121 (7)	
O4	0.1568 (3)	0.3740 (5)	0.7337 (4)	0.0107 (6)	
O5	0.2552 (2)	0.0776 (5)	0.8526 (4)	0.0105 (6)	
O6	0.0652 (2)	0.5022 (5)	0.4280 (4)	0.0104 (6)	

### Atomic displacement parameters (Å^2^)

**Table:**

	*U*^11^	*U*^22^	*U*^33^	*U*^12^	*U*^13^	*U*^23^
Li	0.018 (6)	0.011 (6)	0.020 (7)	0.000	0.012 (6)	0.000
Ho	0.0063 (2)	0.0076 (2)	0.0071 (2)	0.000	0.0034 (1)	0.000
P1	0.0052 (5)	0.0069 (5)	0.0072 (5)	−0.0002 (4)	0.0031 (4)	−0.0001 (4)
P2	0.0051 (5)	0.0072 (5)	0.0073 (5)	−0.0001 (4)	0.0032 (4)	0.0009 (4)
O1	0.012 (2)	0.009 (2)	0.016 (2)	0.001 (1)	0.009 (1)	−0.002 (1)
O2	0.008 (1)	0.012 (2)	0.016 (2)	0.001 (1)	0.008 (1)	0.002 (1)
O3	0.012 (2)	0.014 (2)	0.008 (1)	0.002 (1)	0.004 (1)	0.000 (1)
O4	0.015 (2)	0.007 (1)	0.009 (1)	0.001 (1)	0.007 (1)	0.002 (1)
O5	0.005 (1)	0.016 (2)	0.010 (1)	0.002 (1)	0.005 (1)	0.002 (1)
O6	0.007 (1)	0.014 (2)	0.006 (1)	−0.001 (1)	0.001 (1)	0.000 (1)

### Geometric parameters (Å, °)

**Table:**

Li—O2^i^	1.962 (9)	Ho—O6	2.516 (3)
Li—O2^ii^	1.962 (9)	Ho—O6^iii^	2.516 (3)
Li—O6^iii^	1.999 (9)	P1—O1	1.488 (4)
Li—O6	1.999 (9)	P1—O6	1.499 (3)
Ho—O3^iii^	2.287 (3)	P1—O4	1.589 (4)
Ho—O3	2.287 (3)	P1—O5^vi^	1.594 (3)
Ho—O1^ii^	2.348 (4)	P2—O2	1.486 (3)
Ho—O1^i^	2.348 (4)	P2—O3	1.488 (3)
Ho—O2^iv^	2.411 (3)	P2—O5	1.588 (3)
Ho—O2^v^	2.411 (3)	P2—O4	1.601 (4)
			
O2^i^—Li—O2^ii^	83.7 (5)	O6—P1—O5^vi^	105.0 (2)
O2^i^—Li—O6^iii^	119.57 (14)	O4—P1—O5^vi^	102.7 (2)
O2^ii^—Li—O6^iii^	125.85 (14)	O2—P2—O3	119.6 (2)
O2^i^—Li—O6	125.85 (14)	O2—P2—O5	107.9 (2)
O2^ii^—Li—O6	119.57 (14)	O3—P2—O5	111.9 (2)
O6^iii^—Li—O6	87.2 (5)	O2—P2—O4	104.6 (2)
O1—P1—O6	118.8 (2)	O3—P2—O4	109.7 (2)
O1—P1—O4	106.8 (2)	O5—P2—O4	101.2 (2)
O6—P1—O4	110.8 (2)	P1—O4—P2	131.6 (2)
O1—P1—O5^vi^	111.7 (2)	P2—O5—P1^vii^	134.8 (2)

Symmetry codes: (i) *x*, −*y*+1, *z*−1/2; (ii) −*x*, −*y*+1, −*z*+1; (iii) −*x*, *y*, −*z*+1/2; (iv) −*x*, −*y*, −*z*+1; (v) *x*, −*y*, *z*−1/2; (vi) −*x*+1/2, *y*+1/2, −*z*+3/2; (vii) −*x*+1/2, *y*−1/2, −*z*+3/2.
